# MicroRNA-200s attenuate demyelination caused by *Angiostrongylus cantonensis* in a mouse model by targeting phosphatase and tensin homolog

**DOI:** 10.4103/NRR.NRR-D-24-01112

**Published:** 2025-06-19

**Authors:** Huihui Xiong, Zhixuan Ma, Ge Li, Zhen Niu, Liang Yang, Xiaojie Wu, Liming Wang, Fukang Xie, Chi Teng Vong, Xi Sun, Zhongdao Wu, Ying Feng

**Affiliations:** 1 School of Medicine, South China University of Technology, Guangzhou, Guangdong Province, China; 2 Zhongshan School of Medicine, Sun Yat-sen University, Guangzhou, Guangdong Province, China; 3 Guangdong Provincial Key Laboratory of Pathogenesis, Targeted Prevention and Treatment of Heart Disease, Guangdong Provincial People’s Hospital (Guangdong Academy of Medical Sciences), Southern Medical University, Guangzhou, Guangdong Province, China; 4 State Key Laboratory of Quality Research in Chinese Medicine, Institute of Chinese Medical Sciences, University of Macau, Macao Special Administrative Region, China; 5 Macau Center for Research and Development in Chinese Medicine, University of Macau, Macao Special Administrative Region, China

**Keywords:** *Angiostrongylus cantonensis*, central nervous system, demyelination, endogenous, miR-200s, phosphatase and tensin homolog

## Abstract

Demyelinating diseases of the central nervous system are common, yet few effective strategies for myelin repair and remyelination are available. An increasing number of studies highlight the role of microRNAs (miRNAs) as key regulators of demyelination. miRNA mimics and inhibitors, which are currently in preclinical development, have shown promise as novel therapeutic agents. However, the mechanisms by which they protect myelin are not fully understood. Using a mouse model of acute central nervous system demyelination induced by infection with *Angiostrongylus cantonensis*, we investigated alterations in miRNA expression in the mouse brain. Our findings revealed a significant early-stage increase in the levels of miR-200, particularly miR-200a and miR-200c. Subsequent analysis demonstrated that combined miR-200a and miR-200c overexpression improved neurobehavioral outcomes and attenuated demyelination in *Angiostrongylus cantonensis*-infected mice. Further lipid metabolomic profiling indicated that miR-200a and miR-200c synergistically inhibited the production of phosphatase and tensin homolog (PTEN) and activated the phosphoinositide 3-kinase/protein kinase B/mammalian target of rapamycin signaling pathway, as confirmed by double luciferase reporter assay and western blotting. Additionally, *in vitro* experiments showed that miR-200a and miR-200c protected oligodendrocyte precursor cells from lipopolysaccharide-induced damage and enhanced their survival. Our study indicates the critical role of miR-200a and miR-200c in protecting against central nervous system demyelination by targeting PTEN and modulating key survival pathways. Furthermore, our findings suggest that miR-200a and miR-200c are promising diagnostic biomarkers of and therapeutic targets for treating demyelination-related disorders.

## Graphical abstract

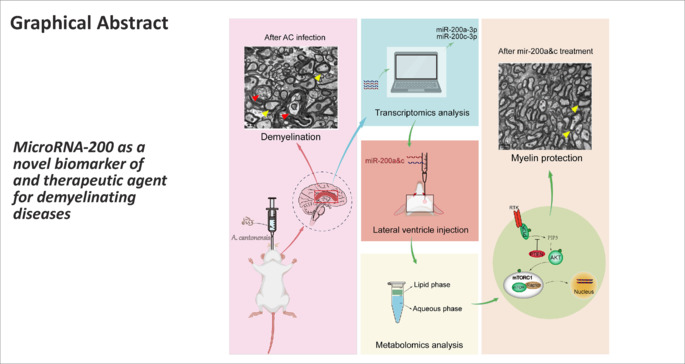

## Introduction

The incidence of central nervous system (CNS) demyelinating diseases, such as multiple sclerosis and neuromyelitis optica, has risen in recent years. The clinical manifestations of these diseases mainly include loss of motor coordination, sensory dysfunction, and cognitive impairment, and they often recur or result in sequelae, seriously affecting patients’ quality of life (Beltrán et al., 2020). The main treatments for demyelinating diseases at present are immune modulators and neuroprotective drugs (Cadenas-Fernández et al., 2021), which have limited efficacy with regards to myelin repair.

MicroRNAs (miRNAs) are a highly conserved class of small non-coding RNAs in eukaryotes and play crucial roles in development, carcinogenesis, stress responses, and viral infections through post-transcriptional gene regulation (Ali Syeda et al., 2020; Hussen et al., 2021; Kapplingattu et al., 2025). miRNAs are increasingly recognized for their involvement in demyelinating diseases. For instance, miR-219 and miR-338 have been implicated in the development of oligodendrocyte lines (OLs) in the mammalian CNS (Milbreta et al., 2019). Within the nervous system, the miR-200 family, which comprises five members, namely miR-200a, miR-200b, miR-200c, miR-141, and miR-429 (Sundararajan et al., 2022), regulates neuronal differentiation and proliferation to promote neurogenesis (Jauhari and Yadav, 2019; Patranabis, 2024) and maintain neuronal survival (Yang et al., 2020; Walker et al., 2022). However, it remains unclear whether miR-200 family members are involved in maintaining the integrity of the myelin sheath within the CNS.

A known target of miR-200s, phosphatase and tensin homolog (PTEN), and its downstream pathway, the phosphoinositide 3-kinase/protein kinase B/mammalian target of rapamycin (PI3K/AKT/mTOR) pathway, are involved in oligodendrocyte progenitor cell (OPC) differentiation and myelination in the CNS. Experimental knockout of mTOR in mouse OLs reduced the number of mature OLs (Wahl et al., 2014). Moreover, persistent AKT overexpression in transgenic mouse OLs or conditional knockout of the PI3K/AKT upstream inhibitor PTEN caused excessive myelination (Ishii et al., 2019). PI3K/AKT/mTORC1 signaling plays a pivotal role in promoting oligodendrocyte differentiation and initiating myelination (Gaesser and Fyffe-Maricich, 2016). However, the role of this pathway in myelin repair and regeneration in demyelinating diseases is not well understood.

*Angiostrongylus cantonensis* (AC), a food-borne zoonotic parasite, is the leading cause of eosinophilic meningitis globally (Cowie et al., 2022; Pandian et al., 2023) and induces severe demyelination in the CNS. In a previous study, we demonstrated that AC-infected mice serve as a valuable model for investigating brain demyelination (Xiong et al., 2021). Here, we examined the effect of miR-200s on AC-induced CNS demyelination.

## Methods

### Animals and *Angiostrongylus cantonensis* infection

Specific pathogen-free BALB/c mice, aged 6–8 weeks and weighing 19–23 g, were purchased from the Laboratory Animal Center of Sun Yat-sen University (Guangzhou, China; license No. SCXK (Yue) 2016-0029). All mice were male to minimize the interference of estrogen. The mice were housed in a barrier environment with individual ventilation cages, six mice per cage, with the ambient temperature maintained at 23°C, humidity at 55%, and a 12-hour light/12-hour dark cycle. *Biomphalaria glabrata* infected with Guangzhou strain AC were obtained from the Department of Parasites, Zhongshan School of Medicine. To retrieve the third-stage larvae, we minced the *Biomphalaria glabrata* and digested the tissue with concentrated HCL and pepsin for about 90 minutes at 37°C. Then, we stopped the digestion with normal saline and picked the active larvae out of the digested tissue under an anatomical microscope. Each mouse was infected with 30 larvae by oral inoculation. This study was approved by the Animal Research Ethics Committee of South China University of Technology (approval No. 2022106) on March 1, 2022 and was conducted in strict accordance with the National Institutes of Health Guide for the Care and Use of Laboratory Animals (8^th^ ed., National Research Council, 2011).

### MiRNA microarray

The mice were randomly divided into groups, with six mice in each group. Mice post-infected with AC on days 2, 7, 14, and 21 and uninfected control mice were anesthetized by intraperitoneal injection with 1% sodium pentobarbital (50 mg/kg, Jiehui Bio, Beijing, China) and then perfused through the cardiac apex. The brains were immediately removed, and total RNA was extracted using TRIzol reagent (Invitrogen, Carlsbad, CA, USA). Total RNA was reverse transcribed to cDNA (miRcute Plus miRNA First-Strand cDNA Kit, TIANGEN BIOTECH, Beijing, China), which was then hybridized to a multiplexed miRNA-specific oligo pool, and the hybrid products were amplified through polymerase chain reaction (PCR). All procedures were performed using the Universal 12 BeadChip (Illumina, San Diego, CA, USA).

### MiRNA fluorescence *in situ* hybridization

Mice post-infected with AC on days 2, 7, 14, and 21 and uninfected control mice were sacrificed as described above, and the brains were removed. The whole brains were fixed in 4% paraformaldehyde and dehydrated in sucrose PBS solutions, then embedded in optimal cutting temperature compound and sliced at a thickness of 10 μm. A specific 5′-digoxigenin-labeled Locked Nucleic Acid (DIG-labeled LNA) probe bound to miRNA-200a-3p was synthesized by GeneBio (Shanghai, China). Anti-digoxigenin-fluorescein and Fab fragments (1:200; Cat# 11207741910, Roche, Basel, Switzerland) were used to detect positive probe signals. To determine whether miR-200a co-localized with the myelin sheath, we performed myelin basic protein (MBP)-labeled staining after hybridization. The brain sections were incubated with a primary rat anti-MBP (Abcam, Cat# ab277483, RRID: AB_277483) antibody overnight at 4°C at a dilution of 1:500, followed by incubation with a secondary antibody (donkey anti-rat) at a dilution of 1:500 (Jackson ImmunoResearch Labs, West Grove, PA, USA, Cat# 712-165-153, RRID: AB_ 2315049) for 1 hour at room temperature (37°C). All procedures were performed as described in previous studies (Tatarakis and Moazed, 2022; Gulanicz et al., 2023). MiRNA fluorescence in situ hybridization (FISH) analysis was performed using ImageJ Fiji (https://imagej.net/software/fiji/).

### RNA preparation and quantitative polymerase chain reaction

Total brain RNA was isolated using TRIzol (Invitrogen) following the manufacturer’s protocol. An miR-X miRNA First-Strand Synthesis Kit (Takara, Shiga, Japan) was used to synthesize the miRNA-200s, while a PrimeScript RT reagent kit (Takara) was used to synthesize the MBP and PTEN cDNAs. A SYBR Premix Ex Taq kit (Takara) was used to perform quantitative PCR (qPCR) for miRNAs and mRNAs using a CFX96 system (Bio-Rad, Hercules, CA, USA). Primers for qPCR were synthesized by Sangon Biotech (Shanghai, China). All kits were used according to the manufacturers’ instructions. Data were analyzed using the 2^–ΔΔCt^ method and normalized toU6. Primer sequences for qPCR are shown in **Table 1**. More detailed methods are presented in **Additional file 1**.

**Table 1:** Primer sequences for quantitative polymerase chain reaction

Primer	Sequence (5'–3')
mmu-miR-200a-3P-F	TAA CAC TGT CTG GTA ACG ATG T
mmu-miR-200b-5P-F	CAT CTT ACT GGG CAG CAT TGG A
mmu-miR-200c-3P-F	TAA TAC TGC CGG GTA ATG ATG GA
mmu-miR-429-3P-F	TAA TAC TGT CTG GTA ATG CCG T
mmu-miR-141-5P-F	CAT CTT CCA GTG CAG TGT TGG A

The internal reference U6 and universal downstream primers for the microRNAs were obtained from a reagent kit.

### Stereotactic injection

To investigate the effect of miR-200 overexpression on myelin in AC-infected mice, the artificially modified mimics agomiR-200a and agomiR-200c were co-injected into the lateral ventricles of mice at 7 days post-infection (dpi). AgomiR-NC was used as a negative control. The agomiRs were all synthesized by GeneBio. To perform the stereotactic injection, the mice were first anesthetized with 1% sodium pentobarbital, and their heads were shaved to expose the surgical area. Erythromycin eye ointment was applied to the mouse corneas to keep them wet. Then, the mice were immobilized on the stereotactic frame via ear bars. A small incision was made in the skin to expose the skull. A burr hole was drilled into the skull at the location of the lateral ventricle (Crawley, 2008) (coordinates: 0.6 mm anterior, 1.5 mm lateral, and 2 mm deep relative to the bregma), and 1 nmol agomiR-200a and 1 nmol agomiR-200c diluted in 4 μL saline, or an equivalent volume of agomiR-NC, was injected into the lateral ventricle using a 22-gauge needle and syringe (Hamilton, Reno, Nevada, USA). The needle was left in place for 3 minutes before being removed to prevent leakage after injection. Finally, the skin was sutured, and lincomycin ointment was applied to the injection site to reduce pain and protect against infection. More detailed methods are presented in **Additional file 1**.

### Neurological accession and brain magnetic resonance imaging

On day 21 post-infection, the mice were weighed, and their motor and sensory functions were assessed using the neurological scoring method described by Parra et al. (2002), in which a higher score indicates better function. We also performed *in vivo* brain magnetic resonance imaging (MRI) using a 7.0 T MR scanner (Bruker BioSpin, MRI GmbH, Ettlingen, Germany) to identify alterations in the brain parenchyma. T1-weighted imaging (T1WI) and T2WI images were collected, as previously described (Xiong et al.,2021). The areas of effusion and corpus callosum lesions were measured using ImageJ, and the ratio of the lesion area within the corpus callosum to the whole brain area was calculated.

### Histopathological observation

Mice were anesthetized by intraperitoneal injection with 1% sodium pentobarbital and then perfused through the cardiac apexwith normal saline. The brains were removed and fixed in 4% paraformaldehyde solution for 24 hours, then dehydrated, cleared, infiltrated, paraffin-embedded, and sectioned into 5-μm-thick slices. Cerebral hemorrhage was detected by hematoxylin and eosin (H&E) staining. Briefly, the paraffin-embedded sections were baked at 65°C for 30 minutes, then dewaxed and rehydrated. They were then stained with hematoxylin for 3 minutes, differentiated in 1% hydrochloric acid ethanol for 3 seconds, and rinsed under running water for 30 minutes to restore the blue color. The sections were then counterstained with 0.5% eosin for 3 minutes. After dehydration and clearing, the sections were mounted with neutral resin. The H&E staining reagents were purchased from Solarbio (Beijing, China). Demyelination was detected using luxol fast blue (LFB) staining. Briefly, the paraffin sections were dewaxed and then immersed in a 0.1% LFB solution for overnight staining at 60°C. After staining, the sections were washed with distilled water and 95% ethanol to remove excess dye. Subsequently, the sections were differentiated in a 0.05% lithium carbonate solution and 70% ethanol. The differentiation reaction was stopped when the gray and white matter were clearly distinguishable under a microscope. Finally, the sections were dehydrated, cleared, and mounted. LFB staining reagents were purchased from Beyotime (Shanghai, China). Demyelination of the corpus callosum ultrastructure was observed by transmission electron microscopy (TEM). The mouse brain was harvested, and the corpus callosum was quickly separated and placed into primary fixative for electron microscopy (2.5% glutaraldehyde and 4% paraformaldehyde). After fixation for 24 hours, the corpus callosum was trimmed to 1 mm × 1 mm × 3 mm under a dissecting microscope, followed by postfixation with osmium tetroxide for 1.5 hours. The tissue was then dehydrated using an ethanol gradient, embedded in epoxy resin, sectioned using an ultramicrotome, and double-stained with uranyl acetate and lead citrate. Finally, demyelination was observed using a 300 kV TEM. The main reagents used for TEM were purchased from Sigma (St. Louis, MO, USA). The inner and outer diameters of the myelinated axons were measured, and the number of myelinated axons in the corpus callosum was counted using ImageJ.

### Western blotting

The corpus callosum was isolated from the mouse brain and snap-frozen in liquid nitrogen. Proteins were lysed by sonication in RIPA lysis buffer (Beyotime) containing protease inhibitor PMSF (1 mg/mL) on ice. The total protein concentration was measured using a NanoDrop One (Thermo Fisher Scientific, Waltham, MA, USA). After dilution in RIPA and protein loading buffer, total protein was denatured by incubation in a water bath at 98°C for 10 minutes. A 5% stacking gel was prepared, and 30 µg of protein per lane was separated by electrophoresis on a 10% resolving gel (Beyotime). The proteins were then transferred to a PVDF membrane (Millipore, Billerica, MA, USA). The membrane was blocked with 5% skim milk (BD Difco, Franklin Lakes, NJ, USA) or 5% bovine serum albumin (Solarbio) at room temperature for 1 hour and then incubated with primary antibodies overnight at 4°C. Following 15-minute washes × 3 with TBST, the membrane was incubated with HRP-conjugated secondary antibodies at room temperature for 1 hour. Chemiluminescent signals were detected using an ECL kit (Bio-Rad). The antibodies used were as follows: rabbit anti-PTEN (1:10,000, Abcam, Cambridge, UK, Cat# ab267787, RRID: AB_2923364), rabbit anti-PI3K (1:1000, CST, Boston, MA, USA, Cat# 4292, RRID: AB_329869), rabbit anti-AKT (1:1000, CST, Cat# 4685, RRID: AB_ 2069866), p-AKT (1:1000, CST, Cat# 4060, RRID: AB_ 2315049), mTOR (1:1000, CST, Cat#2983; RRID: AB_ 2105622), p-mTOR (1:1000, CST, Cat# 2971, RRID: AB_ 330970), rabbit anti-p-PI3K (1:1000, Bioworld, Dublin, OH, USA, Cat# BS3006, RRID: AB_ 1663719), rat anti-MBP (1:5000, Abcam, Cat# ab277483, RRID: AB_277483), rabbit anti-β-actin (1:5000, Proteintech, Rosemont, IL, USA, Cat# 66009-1-I, RRID: AB_268793), goat anti-rabbit conjugated to HRP (1:10,000, CST, Cat# 7074, RRID: AB_2099233), and goat anti-rat conjugated to HRP (1:10,000, Proteintech, Cat# SA00001-6, RRID: AB_ 2864369). The optical densities of the bands were measured using ImageJ software. PTEN and MBP protein bands were normalized to β-actin, while p-PI3K, p-AKT, and p-mTOR were normalized to the corresponding non-phosphorylated proteins.

### Primary oligodendrocyte progenitor cell culture

To obtain neural stem cells (NSCs), the hippocampus was isolated from 1- to 3-day-old BALB/c mice. After removing the overlying meninges and blood vessels, the hippocampus was was cut into small pieces and placed in DMEM/F12 medium (Gibco, Carlsbad, CA, USA) containing 20 ng/mL basic fibroblast (R&D system, Minneapolis, MN, USA) and 1% B27 supplement (Gibco) at 37°C with 5% CO_2_. After 5–7 days of culture, a large number of clonal spheres formed and were characterized as NSCs by their nestin-positive staining. The NSC spheres were pipetted up and down to generate a single-cell suspension, which was seeded into poly-D-lysine-coated (Sigma) culture flask containing induction medium consisting of 30 ng/mL T3 (Sigma) and 10 ng/mL Platelet-Derived Growth Factor-AA (PDGF-AA) (Peprotech, Cranbury, NJ, USA). A large number of cells that included OPCs and astrocytes emigrated from the clonal spheres after 1–2 days of culture. These cells were dissociated by treatment with 0.125% trypsin for 2–3 minutes at 37°C and then seeded into a new coated culture flask. After 20–40 minutes, during which time the astrocytes adhered to the flask, the culture medium was collected and seeded again into a new coated culture flask. This process was repeated twice to obtain purified OPCs, which were then cultured in DMEM/F12 medium containing 1% B27, 1% N_2_ (Gibco), 20 ng/mL PDGF-AA, 20 ng/mL insulin-like growth factor 1 (R&D system), 40 ng/mL T3, and 5% FBS (PAN-Biotech, Bavaria, Germany).

### Immunofluorescence staining

The brain tissues were cryosectioned to a thickness of 10 μm. The sections were treated with PBS containing 1% Triton X-100 for 30 minutes and then washed with PBS for 15 minutes (three times). Subsequently, they were blocked with a blocking solution (PBS containing 5% BSA, 10% donkey serum, and 0.3% Triton X-100) at room temperature for 1 hour. Cultured cells were fixed with 4% paraformaldehyde at room temperature for 15 minutes, washed with PBS for 15 minutes (three times), and then blocked with PBS containing 5% BSA and 0.3% Triton X-100 at room temperature for 1 hour. Both tissue sections and cells were incubated with primary antibodies diluted in their respective blocking solutions overnight at 4°C. After washing with PBS three times for a total of 15 minutes, they were incubated with fluorescently labeled secondary antibodies at room temperature in the dark for 1 hour. The sections were incubated with 4′,6-diamidino-2-phenylindole for 5 minutes before mounting, and the cells were directly observed under a fluorescence microscope. The primary antibodies used were as follows: rabbit anti-CNPase (2′,3′-cyclic nucleotide 3′-phosphodiesterase) (CC-1; 1:200, Millipore, Cat# OP80, RRID: AB_2057371), mouse anti-glial fibrillary acidic protein (1:500, CST, Cat# 3670, RRID: AB_561049), mouse anti-nestin (1:200, Millipore, Cat# MAB353), rabbit anti-NG2 (1:200, Millipore, Cat# AB5320, RRID: AB_11213678), rabbit anti-MBP (1:200, Abcam, Cat# PC35101, RRID: AB_40390) and rabbit-anti oligo2 (1:200, Millipore, Cat# AB9610, RRID: AB_570666). The secondary antibodies used were as follows: donkey anti-rabbit conjugated to Alex Fluor 488 (1:500, Jackson ImmunoResearch, Cat# 711-545-152, RRID: AB_2313584) and donkey anti- mouse conjugated to Cy3 antibody (1:500, Jackson ImmunoResearch, Cat# 715-165-150, RRID: AB_2340813). Immunofluorescence data analysis was performed using ImageJ.

### Dual-luciferase reporter assay

Binding sites between miR-200a and PTEN and between miR-200c and PTEN were predicted using TargetScan (https://www.targetscan.org/vert_80/). Plasmids containing wild-type and mutant PTEN sequences were constructed by GenePharma (Shanghai, China). The negative control, miR-200a, and miR-200c AgomiRs used in this experiment were the same as those used in the stereotactic injection experiment. Purified OPCs (5 × 10^3^ cells/well) were seeded into 96-well plates and cultured at 37°C and 5% CO₂ for 24 hours. Subsequently, the cells were transfected with 0.1 μg of the plasmids and 50 nM of the miRNAs per well using lipofectamine 3000 (Invitrogen). After 48 hours, relative fluorescence was measured using a dual-luciferase reporter assay system (E1910, Promega, Madison, WI, USA) and a microplate reader (TECAN, Infinite F500, Grödig, Salzburg, Austria), according to the standard methods.

### Cell Counting Kit-8 Assay

Purified OPCs were seeded into 96-well plates at a density of 5 × 10^3^ cells/mL and cultured for 24 hours. Three concentrations (10, 50, and 100 nmol/mL) of agomir-200a and agomir-200c were added. AgomiR-200a and agomiR-200c were co-transfected into OPCs using Rector (GeneBio). The transfection medium was replaced with fresh OPC culture medium after 6 hours of incubation. At 24 hours after transfection, 5 μg/mL of lipopolysaccharide (LPS) (Invitrogen) was added to each well and allowed to stimulate the OPCs for 24 hours. Subsequently, 10 μL of Cell Counting Kit-8 solution (Dojindo, Kumamoto, Japan) was added to each well, and the plates were reincubated at 37°C for 2 hours. Then, the absorbance was measured at 450 nm using a microplate reader (TECAN).

### Statistical analysis

Statistical analyses were performed using IBM SPSS Statistics 22.0 (IBM Corp, Armonk, NY, USA). GraphPad Prism (version 8.0, GraphPad Software, San Diego, CA, USA) was used to create the graphs. Data are presented as mean ± standard deviation (SD) or standard error of the mean (SEM), and most statistical analyses were carried out using one-way analysis of variance with Bonferroni *post hoc* correction for statistical evaluation of more than two groups. The Mann–Whitney *U* test was used to perform the median difference test on the g-ratio of myelinated axons in the corpus callosum. Statistical significance was set at *P* < 0.05.

## Results

### MiR-200 expression is markedly increased in the mouse brain in the early stage of *Angiostrongylus cantonensis* infection

**Figure 1:**
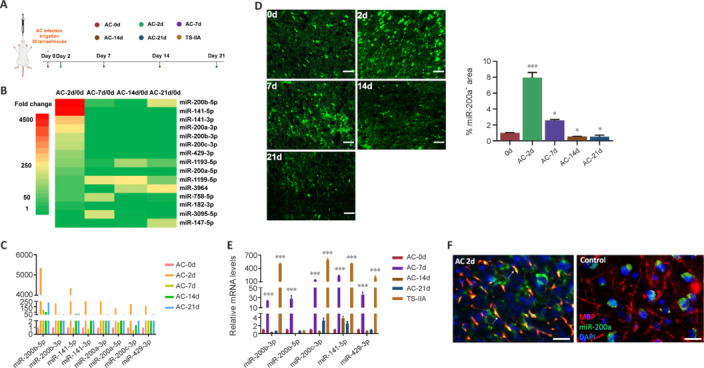
miR-200 expression was markedly increased in the brains of mice infected with *Angiostrongylus cantonensis*. (A) Timeline of the animal experiment. Mice infected with AC were sacrificed at 2, 7, 14, and 21 days post-infection, and brain tissues were obtained for microarray, fluorescence *in situ* hybridization (FISH), and quantitative polymerase chain reaction. Uninfected healthy mice were used as controls. (B) Heatmap of some miRNAs that were highly expressed in the infected group compared with the control group. (C) Bar chart of the heat map statistics. (D) FISH analysis showing miR-200a expression in the brains of mice infected with AC. Scale bars: 50 μm. Green fluorescence indicates miR-200a. (E) miR-200 expression levels in the brains of mice infected with AC and those treated with TS-IIA, as verified by quantitative polymerase chain reaction. (F) miR-200a expression in the myelin sheath was markedly increased 2 days after AC infection (white arrows). Scale bars: 25 μm. The white arrows indicate co-localization. Data are expressed as mean ± SEM (*n* = 3 animals/group). *P < 0.05, ***P < 0.001, *vs.* control group. AC: *Angiostrongylus cantonensis*; miR: microRNA; TS-IIA: Tanshinone IIA.

Dynamic changes in miRNA expression levels in the brains of AC-infected mice were assessed by microarray assay at 0, 2, 7, 14, and 21 dpi (**Figure 1A**). The results revealed a significant upregulation in miR-200 expression at 2 dpi, followed by a decrease at 7 dpi, and finally a decrease to basal level at 14 dpi (**Figure 1B** and **C**). This trend was corroborated by miR-200a FISH (**Figure 1D**). On the basis of our previous findings demonstrating attenuation of demyelination in AC-infected mice treated with Tanshinone IIA (TS-IIA) (Feng et al.,2019), we treated mice with TS-IIA and investigated miR-200 miRNA expression levels. All miR-200 family members except miR-200b exhibited greater expression in the TS-IIA–treated group than in the negative control group, as well as greater expression than in the infected group at 7 dpi. Notably, miR-200c-3p and miR-200a-3p exhibited the highest expression levels (**Figure 1E**). Consequently, the roles of miR-200a and miR-200c in AC-induced CNS demyelination were further investigated. To determine the relationship between miR-200s and myelin, brain sections from AC-infected mice were co-stained for miR-200a and MBP. The results showed that miR-200a and MBP co-localized, and their expression levels were markedly increased in the myelin sheath at 2 dpi (**Figure 1F**).

### Combined overexpression of miR-200a and miR-200c reduces demyelination in *Angiostrongylus cantonensis*-infected mice

**Figure 2:**
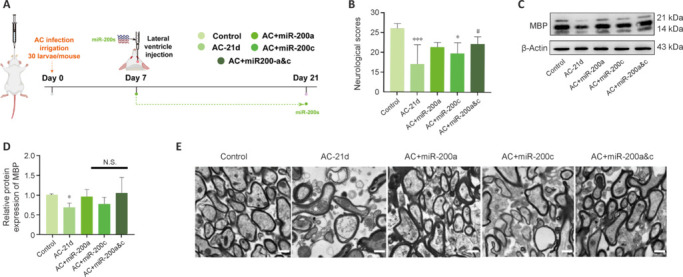
Combined application of miR-200a and miR-200c has a stronger effect than application of either miRNA alone on demyelination in AC-infected mice. (A) Timeline of the animal experiment. Mice infected with AC were injected with miR-200s at 7 dpi and sacrificed at 21 dpi. (B) Neurological scores in the different groups. *n* = 6. (C, D) Myelin-associated protein (MBP) expression levels in the corpus callosum. *n* = 3. (E) Myelin sheaths in the corpus callosum observed by transmission electron microscopy. Scale bars: 1 μm. Data are expressed as mean ± SD. *P < 0.05, ***P < 0.001, *vs*. control group; #*P* < 0.05, *vs.* AC-21 d group. AC: *Angiostrongylus cantonensis*; miR: microRNA; N.S.: not significant.

Given the aforementioned results showing that miR-200a and miR-200c may be significant in AC infection, we next asked whether they exert their effects separately or synergistically. miR-200a and miR-200c were administered to AC-infected mice at 7 dpi to maintain high expression levels of both miRNAs in the mouse brain (**Figure 2A**). Compared with the AC-infected group at 21 dpi, combined administration of miR-200a and miR-200c increased the neurological scores of the mice (*P* < 0.05; **Figure 2B**). TEM and western blotting indicated that combined administration of miR-200a and miR-200c caused a more significant reduction in myelin sheath damage than administration of either miRNA alone (**Figure 2C–E**). When the mice were treated with miR-200a and miR-200c inhibitors, fatality and disability rates did not differ significantly from those in the AC infection group, despite notable damage to myelin sheaths in the corpus callosum in the treated group (data not shown).

**Figure 3:**
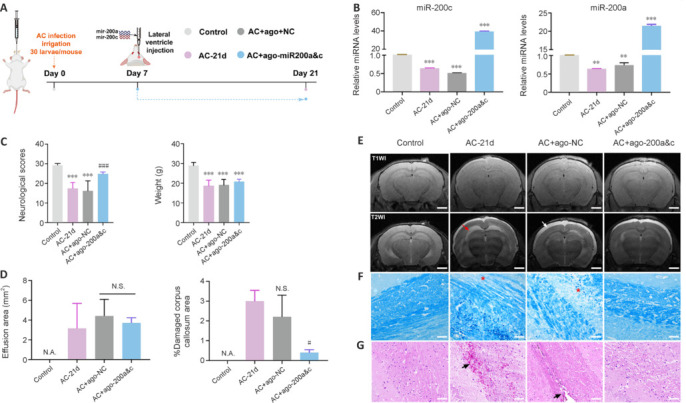
Neurological outcomes in AC-infected mice were improved by treatment with miR-200s. (A) Timeline of the animal experiment. AC-infected mice were co-injected with agomiR-200a and -200c or agomiR-NC in the lateral ventricle on day 7 post-infection. Two weeks later, the neurological status of the mice was assessed, magnetic resonance imaging was performed, and brain tissue was harvested for subsequent experiments. (B) miR-200c and miR-200a expression levels were quantified by quantitative polymerase chain reaction, using U6 as the internal reference. *n* = 3. (C) Left: mouse neurological scores; right: mouse weights. (D) Left: area of cerebral effusion; right: area of damaged corpus callosum. *n* = 3. (E) Representative MRI images. Red arrows indicate corpus callosum, and white arrows indicate subdural effusion. Scale bars: 2 mm. (F) Luxol fast blue (LFB) staining of the corpus callosum. Red asterisks indicate a decrease in the number of myelin sheaths. Scale bars: 20 μm. (G) Hematoxylin and eosin staining of the corpus callosum. Black arrows indicate hemorrhage. Scale bars: 50 μm. Data are expressed as mean ± SD. *P < 0.05, ***P < 0.001, *vs.* control group; #*P* < 0.05, *vs*. AC-21 d group. AC: *Angiostrongylus cantonensis*; miR: microRNA; N.A.: not applicable; N.S.: not significant; T1WI: T1-weighted imaging; T2WI: T2-weighted imaging.

Subsequently, we successfully overexpressed miR-200a and miR-200c in AC-infected mice (**Figure 3A** and **B**). The neurological scores of the infected and NC groups were significantly lower than those of the control group. Despite an overall decrease in animal weight following AC infection (including in the miR-200 injection group), the neurological scores in the overexpression groups were significantly higher than those in the AC-21 d group (*P* < 0.001; **Figure 3C**). T1WI and T2WI coronal MRI of the control mouse brain clearly showed the subdural space, and the corpus callosum had a lower density signal than the cortex (**Figure 3E**). In the infected group, T2WI showed high corpus callosum signals (**Figure 3E**), which is consistent with the findings reported by Wang et al. (2019) for the cuprizone toxin model and those reported by Thomas et al. (2020) for the EAE model, indicating corpus callosum injury. In addition, the subdural space was not apparent in the infected mice, and T1WI and T2WI showed intense white signals around the brain parenchyma surface (**Figure 3E**), which is consistent with MRI findings from AC-infected dogs (Wun et al., 2021) and AC-infected rats (Shyu et al., 2014). The substantial subdural effusion that we observed was an inflammatory exudate owing to the eosinophilic meningitis caused by AC. The damage to the corpus callosum was much less in the agomiR-200a and agomiR-200c groups compared with the infected group, but there was no discernible difference in effusion among the three groups (**Figure 3D** and **E**). This finding suggests that miR-200a and miR-200c attenuate demyelination but have no effect on eosinophilic meningitis. LFB staining of brain tissue from the agomiR-200a and agomiR-200c groups showed less demyelination compared with the infected group (**Figure 3F**), and H&E staining showed reduced AC-induced hemorrhage in the corpus callosum in the agomiR-200a and agomiR-200c groups compared with the negative control group (**Figure 3G**).

**Figure 4:**
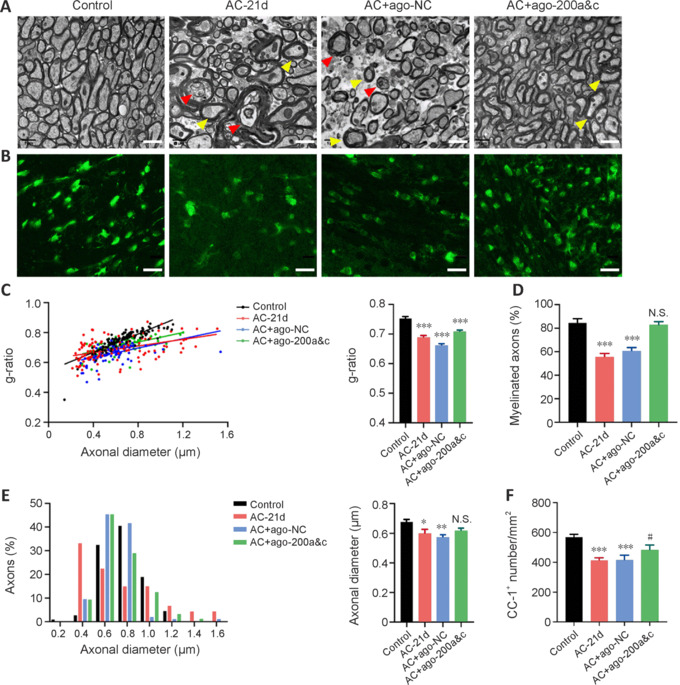
Combined miR-200a and miR-200c overexpression reduces demyelination in AC-infected mice. (A) The ultrastructure of the corpus callosum was observed by transmission electron microscopy. Red arrowheads indicate damaged myelinated axons, and yellow arrowheads indicate thickened myelin. Scale bars: 1 μm. (B) Immunofluorescence analysis of CC-1–labeled cells (green) in the corpus callosum. Scale bars: 20 μm. (C–E) Statistical analysis of the g-ratio, diameter, and ratio of myelinated axons. Data are expressed as mean ± SEM. *n* = 3 animals/group. (F) Statistical analysis of positive CC-1 staining in the corpus callosum. *n* = 3 animals/group. Data are expressed as mean ± SD. *P < 0.05, **P < 0.01,***P < 0.001, *vs*. control group; #*P* < 0.05, *vs.* AC-21 d group. AC: *Angiostrongylus cantonensis*; miR: microRNA; N.S.: not significant.

TEM showed that most axons in the corpus callosum of mice in the control group were myelinated, and the sheath fibers were tightly arranged, with a g-ratio of 0.752 ± 0.065 (**Figure 4A**, **C**, and **D**). Compared with the control group, both the number of myelinated axons and the g-ratio were significantly decreased in the infected group (0.688 ± 0.075; **Figure 4C** and **D**), which is consistent with the observations made by Xiu et al. (2017) in a cuprizone-induced demyelination model. In addition, demyelinated fragments and abnormal thickened myelin were observed (**Figure 4A**). There was no significant difference in axon myelination or distribution of axon diameters between the miR-200a and miRNA-200c overexpression and control groups (**Figure 4A**, **D**, and **E**). However, the g-ratio in the overexpression group was lower than that in the control group (**Figure 4C**). We also determined the number of oligodendrocytes in the corpus callosum. Immunofluorescence staining showed no difference in the number of CC-1-positive cells, indicating oligodendrocytes, in the corpus callosum of the overexpression group compared with the control group (**Figure 4B** and **F**). These data indicate that miR-200 overexpression reduced demyelination and promoted oligodendrocyte survival.

### miR-200s regulate myelin basic protein expression by inhibiting PTEN, thereby activating the PI3K/AKT/mTOR signaling pathway, in *Angiostrongylus cantonensis*-infected mice

**Figure 5:**
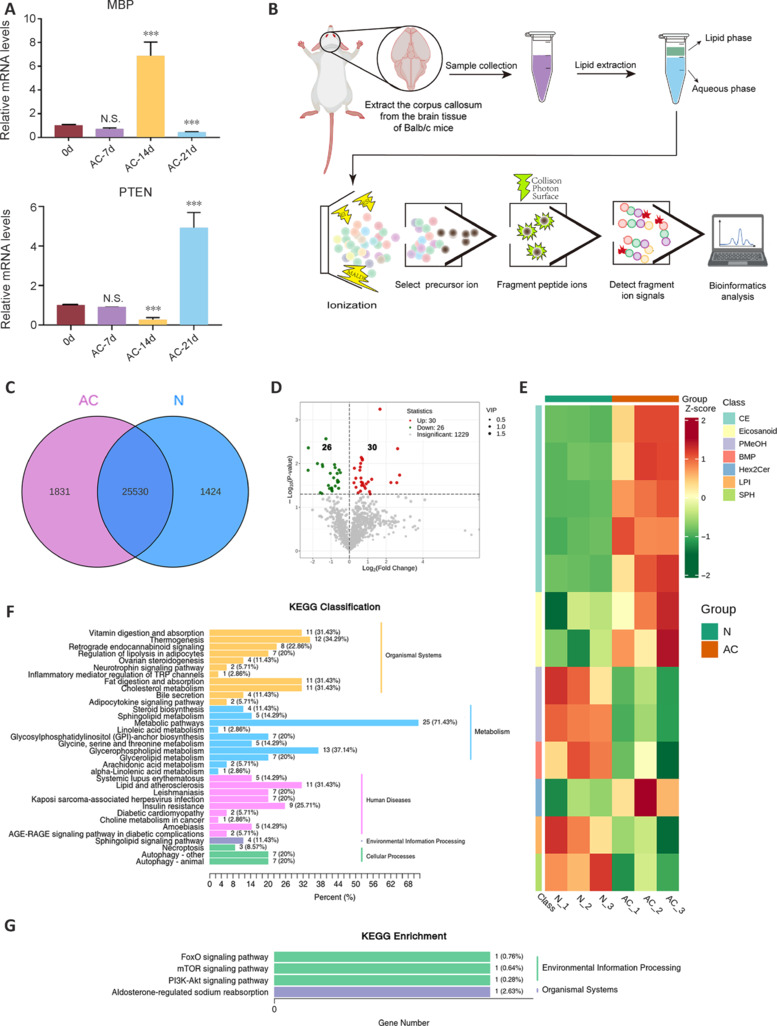
Possible molecular mechanisms by which AC infection causes brain demyelination. (A) MBP and PTEN mRNA levels in the brains of AC-infected mice. GAPDH was used as an internal reference. (B) Targeted quantitative proteinomics analysis workflow. Proteins were purified from the brains of BALB/c mice (normal and AC-infected), then reduced and enzymatically cleaved to generate peptides. Next, all peptide fragments were ionized. Subsequently, mother ions specific to known multiple sclerosis target molecules were selected. Then, collision-induced fragmentation of mother ions was performed to remove interference from other ions. Finally, mass spectrometry signals were collected and bioinformatic analysis was performed for specific ions. (C) Venn diagram of genes associated with lipid metabolism in the AC-infected and normal groups. (D) Volcano plot of gene expression levels. (E) Heatmap of upregulated and downregulated genes. (F) Kyoto Encyclopedia of Genes and Genomes (KEGG) analysis showing the effect of AC infection on organ formation, metabolism, human diseases, and some cellular processes. (G) Further KEGG enrichment analysis indicated potential major influences of AC infection on the mammalian target of rapamycin (mTOR) and PI3K/AKT pathways. AC: *Angiostrongylus cantonensis*; GAPDH: glyceraldehyde-3-phosphate dehydrogenase; MBP: myelin basic protein; PI3K: phosphoinositide 3-kinase; PTEN: phosphatase and tensin homolog.

Compared with the control group, MBP mRNA expression in the brains of infected mice was significantly increased at 14 dpi and decreased at 21 dpi, which is consistent with Lin et al. (2010). This indicates that myelin repair occurs after AC infection, but is ultimately unsuccessful. PTEN mRNA expression exhibited an opposite expression pattern to MBP mRNA, in that it was decreased at 14 dpi and increased at 21 dpi (**Figure 5A**). These results suggest a potential relationship between MBP and PTEN expression levels. To explore the possible molecular mechanisms of AC-induced brain demyelination, we conducted a lipid metabolomic analysis of brain tissues from normal and infected mice (**Figure 5B**). The results revealed 1831 and 25,530 differentially and similarly regulated genes, respectively, in brains of infected mice compared with in the brains of normal mice (**Figure 5C**). Further analysis of the differentially regulated genes revealed 30 significantly upregulated genes, with prominent enrichment of cholesterol ester (CE), the icosanoid bioactive molecule set, and Hex2Cer clusters. Phosphatidylmethanol, bone morphogenetic protein, lysophosphatidylinositol, and sphingolipids were the most significantly downregulated metabolites (**Figure 5D** and **E**). Kyoto Encyclopedia of Genes and Genome (KEGG) classification indicated that the above gene regulatory processes are related to organ formation, metabolism, human diseases, and some cellular processes. Following AC infection, the altered molecules were primarily concentrated in metabolic pathways (**Figure 5F**). Furthermore, KEGG enrichment analysis suggested that the mTOR and phosphatidylinositide 3-kinases-serine/threonine kinase (PI3K/AKT) pathways might be crucial to demyelination (**Figure 5G**).

**Figure 6:**
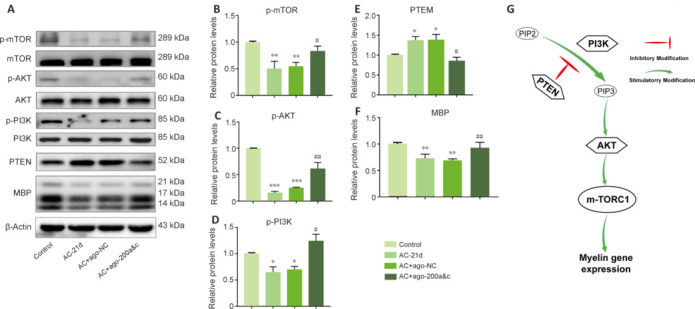
miR-200s regulated MBP expression by inhibiting PTEN, thereby activating PI3K/AKT/mTOR signaling, in AC-infected mice. (A) Representative western blotting images of protein expression in the corpus callosum. (B–F) Quantification of the data shown in A. PTEN and MBP expression levels were normalized to β-actin, and the expression levels of the phosphorylated proteins were normalized to their non-phosphorylated counterparts. Data are expressed as mean ± SEM (*n* = 3 animals/group). *P < 0.05, **P < 0.01, ***P < 0.001, *vs.* control group; #*P* < 0.05, ##*P* < 0.01, *vs*. AC-21 d group. (G) Schematic diagram of miR-200-mediated promotion of myelin formation through the PI3K/AKT/mTOR signaling pathway. AC: *Angiostrongylus cantonensis*; GAPDH: glyceraldehyde-3-phosphate dehydrogenase; MBP: myelin basic protein; mTOR: mammalian target of rapamycin; PI3K: phosphoinositide 3-kinase; PTEN: phosphatase and tensin homolog.

To further investigate the mechanism of action of miR-200s in the myelin sheath in AC-infected mice, we examined the expression of components of the PTEN/PI3K/AKT/mTOR pathway by western blotting. Compared with the control group, PTEN expression was significantly increased (*P* < 0.05) and MBP, PI3K, AKT, and TOR expression levels significantly decreased (all *P* < 0.01) in the AC-infected 21 d group. The opposite expression trends were seen in the overexpression group (**Figure 6A–F**). These results strongly imply that miR-200s directly interact with the PTEN/PI3K/AKT/mTOR pathway and regulate MBP expression by inhibiting PTEN to activate PI3K/AKT/mTOR signaling in AC-infected mice (**Figure 6G**).

### miR-200s protect oligodendrocyte progenitor cells *in vitro* by targeting PTEN

**Figure 7:**
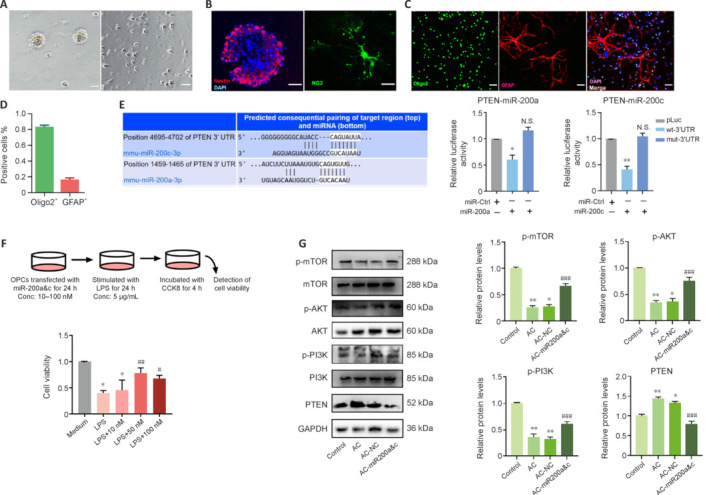
miR-200s target PTEN and protect OPCs from LPS-induced injury. (A) Left, primary neural stem cells (NSCs); right, primary OPCs. (B) Nestin and NG2 were used to identify NSCs and OPCs, respectively. (C, D) Oligo2 was used to identify OPCs, and GFAP was used to identify astrocytes. The percentages of these two cells indicate the purity of the OPC population. Scale bars: 50 μm in A–C. (E) miR-200a/-200c binding sites predicted using the Target Scan database. (F) The wild-type or mutant reporter plasmid was co-transfected with miR-200a/-200c or miR-NC into OPCs. (G) Representative western blot and quantification of mTOR, AKT, PI3K, and PTEN expression. Data are expressed as mean ± SEM (*n* = 3 animals/group). *P < 0.05, **P < 0.01, *vs*. control group (pLuc); ###*P* < 0.001, *vs.* AC-21 d group or LPS group. AC: *Angiostrongylus cantonensis*; GAPDH: glyceraldehyde-3-phosphate dehydrogenase; LPS: lipopolysaccharide; MBP: myelin basic protein; mTOR: mammalian target of rapamycin; OPCs: oligodendrocyte progenitor cells; PI3K: phosphoinositide 3-kinase; PTEN: phosphatase and tensin homolog.

To determine whether PTEN is an miR-200 target gene, we investigated possible miR-200a and miR-200c binding sites in PTEN using the TargetScan database. Dual-luciferase reporter assays were performed on OPCs to verify the potential binding sites. We successfully induced NSCs to differentiate into OPCs, and the OPC purity exceeded 80% (**Figure 7A–D**). Co-transfection of the wild-type reporter plasmid with agomiR-200a or agomiR-200c caused a decreased in fluorescence; however, there was no difference in fluorescence levels between the mutant group and the control group. miR-200a and miR-200c targeted PTEN. The miR-200c binding site is in the PTEN 3′-UTR at residues 4695–4702, while the miR-200a binding site is in the PTEN 3′-UTR at residues 1459–1465 (**Figure 7E**).

To verify the protective effects of miR-200a and miR-200c on OLs *in vitro*, agomiR-200a and agomiR-200c were co-transfected into OPCs, which were treated with LPS to mimic *in vivo* injury (Yao et al., 2010; Peymani et al., 2018). A Cell Counting Kit-8 was used to assess OPC survival. Compared with the LPS-treated group, treatment with miR-200s increased the viability of LPS-treated OPCs, with the most significant increase observed in OPCs treated with the miR-200s at a concentration of 50 nM (**Figure 7F**). Next, primary OPCs derived from BALB/c mice were pre-treated with miR-200a and miRNA-200c mimics. The OPCs were treated with AC antigen to mimic *in vivo* injury (refer to the supplementary material for more information). Subsequently, the expression levels of PTEN/PI3K/AKT/mTOR signaling pathway components in the control and miR-200a and miR-200c treatment groups were assessed by western blotting. PI3K/AKT/mTOR expression levels were increased, whereas that of PTEN was decreased, in the treatment groups compared with the control group (**Figure 7G**). Immunofluorescence staining showed that there were more NG2-positive cells in the overexpression group than in the infection group (**Additional Figure 1**). These data indicate that miR-200 overexpression reduced demyelination and promoted oligodendrocyte survival. These results were consistent with those from the *in vivo* experiments, further demonstrating that miRNA-200a and miRNA-200c primarily activate the PI3K/AKT/mTOR signaling pathway by inhibiting PTEN during AC infection.

### miR-200s attenuate demyelination by improving lipid metabolism levels

**Figure 8:**
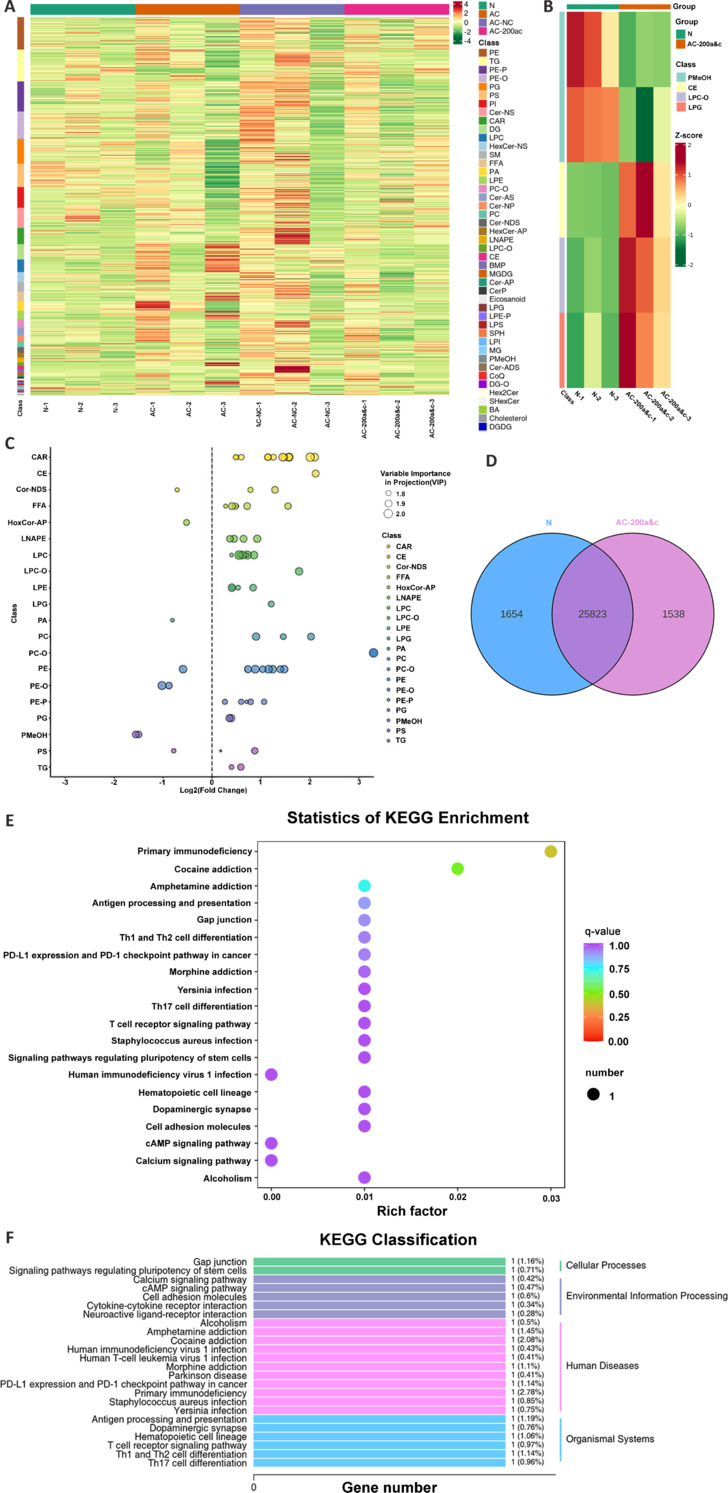
MiR-200s attenuate demyelination by modulating lipid metabolism levels. (A) Heatmap of genes associated with lipid metabolism in the normal, AC-infected, AC-NC, and AC-200ac groups. (B) Heatmap of genes encoding crucial lipid metabolism factors that were differentially expressed in the normal and AC-200ac groups. (C) Bubble plot displaying the upregulation and downregulation of key lipid metabolism gene modules that were differentially expressed in the AC-200ac group compared with the normal group. (D) Venn diagram of genes in the normal and miR-200ac treatment groups with similar expression trends. (E) Kyoto Encyclopedia of Genes and Genome (KEGG) enrichment analysis showing genes in the AC-200ac group related to primary immunodeficiency, cocaine addiction, and amphetamine addiction. (F) KEGG clustering analysis showing the primary signaling pathways involved in normal physiological processes in neural cells. AC: *Angiostrongylus cantonensis*.

To confirm the protective effects of miR-200a and miR-200c against AC-induced brain demyelination, we performed a detailed analysis of lipid metabolism in the control, infected, AC-NC, and AC-200a and AC-200c groups. We found that the levels of glycerolipids and glycerophospholipids, including lysophosphatidylcholine, lysophosphatidylethanolamine, phosphatidic acid, and free fatty acids, increased significantly following AC infection but declined to near-normal levels after miR-200a and miR-200c treatment (**Figure 8A**). Glycerophospholipids are essential components of cell membranes and are crucial for protein recognition and signal transduction on the cell membranes. Only relatively minor changes were observed in phosphocholine and LPS levels among the groups (**Figure 8A**). The miR-200a and miRNA-200c groups exhibited similar protein expression profiles to the normal group (**Additional Figure 2**). Similar trends in protein expression were observed compared with the normal group; however, the normal group had more significant differential clusters (**Figure 8B**), and its CE cluster molecules were downregulated compared with those in the infected group. In total, there were 25,823 genes with similar expression trends between the AC-200a and AC-200c group and the normal group (**Figure 8D**), indicating a significant therapeutic effect of miR-200a and miR-200c on lipid metabolism (**Figure 8C**). KEGG classification and enrichment analysis suggested that miR-200a and miR-200c treatment affected signaling pathways related to stem cell pluripotency, cell adhesion molecules, and neuroactive ligand–receptor interactions (**Figure 8E** and **F**). These results further underscore the critical role of miR-200a and miR-200c treatment at the lipid metabolism level, which is a crucial factor in mediating the mTOR and PI3K/AKT pathways, leading to the attenuation of demyelination after brain injury.

## Discussion

Current strategies for treating CNS myelin disorders largely focus on oligodendrocyte transplantation to promote myelin sheath formation at injury sites (Feng et al., 2023). Recent studies have indicated that miRNAs hold significant promise for regulating OPC growth, differentiation, and development (Zhao et al., 2022; Perdaens et al., 2024; Qiu et al., 2024). MicroRNAs (miRNAs) play a critical role in the central nervous system, particularly in neuronal development, disease, and injury (Ma et al., 2023). Notably, miRNAs are active in the gonadotropin-releasing hormone network within the hypothalamus. Among these, miR-200/429 and miR-155 have been identified as key regulators of a developmental switch that controls gonadotropin-releasing hormone promoter activity (Messina et al., 2016), which initially drew our attention to the miR-200 family. In our previous research on the therapeutic effects of tanshinone IIA in demyelinating conditions, we found that tanshinone significantly suppressed pro-inflammatory cytokines such as interleukin-16, interleukin-1β, and tumor necrosis factor-α, while upregulating several miRNAs. Specifically, miR-200a-3p and miR-200c-3p were markedly elevated during the early phase of infection, returning to baseline levels later. Both miRNAs contributed significantly to the attenuation of central nervous system demyelination (Feng et al., 2019). In our study, we used miR-200a and miR-200c to attenuate AC-induced demyelination and uncovered a novel mechanism by which these miRNAs protect OPCs by modulating lipid metabolism.

An acute demyelination model was established through oral administration of third-stage AC larvae to BALB/c mice, which caused significant CNS demyelination and neurobehavioral changes in the mice after 21 days. Short-term infections (3–4 weeks) reportedly precipitate significant demyelination (Lin and Lai, 2009; Xiong et al., 2021). The neurobehavioral scoring scale used in this study was adapted from Parra et al. (2002) and provides a more detailed and comprehensive method to evaluate changes in neural function than the five-point scale (Huntemann et al., 2022), which mainly evaluates tail tension and muscle tension. Our results confirmed successful establishment of an AC-induced acute demyelination model, in which demyelination was alleviated by miR-200 overexpression.

In recent years, detecting miRNA-based biomarkers in biological fluids has emerged as a promising approach for early diagnosis of multiple sclerosis, offering valuable insight into clinical subtypes and potential treatment modalities (Muñoz-San Martín et al., 2022). Preclinical studies have developed miRNA mimetics and inhibitors that demonstrate promising therapeutic effects. For example, one study analyzing the expression of astrocyte-derived exosome miRNAs purified from the serum of patients with optic neuromyelitis spectrum disorder confirmed that serum exosome miR-129-2-3p levels were significantly elevated in patients with NMOSD compared with healthy individuals and correlated with disease severity (Xie et al., 2023). On this basis, we analyzed the miRNA expression profiles of brain tissue from AC-infected mice to identify potential candidates for repairing CNS demyelination.

In patients with MS, miR-200 is a biomarker of disease progression (Muñoz-San Martín et al., 2022). Our transcriptomic analysis revealed significant upregulation of miR-200a and miR-200c expression levels at 2 dpi, followed by a decrease at 7 dpi, and finally decreasing to basal level at 14 dpi. Jin et al. (2012) observed similar dynamic changes in miR-200a and miR-200c levels in the cortex and striatum in an animal model of Huntington’s disease. Similarly, Naghavian et al. (2015) reported upregulation of miR-141 and miR-200a levels in the blood of patients with recurrent MS, which supports our findings.

Despite these findings, evidence to substantiate the therapeutic efficacy of miR-200s in demyelination is limited. Therefore, we overexpressed miR-200s to assess their therapeutic effects in other demyelinating diseases. The combination of miR-200a and miR-200c yielded superior results, as evidenced by improved neurobehavioral scores in the experimental group and reduced mortality, and mitigated the pathological changes in the myelin sheath among the infected mice. Nevertheless, the g-ratio of myelinated axons in mice treated with these miRNAs was still lower than that seen in the control mice. This may be related to the early stage of myelin repair (Ineichen et al., 2021). The correlation (g-ratio) between axon diameter and myelin thickness is established during myelination development, which often results in an abnormal g-ratio after remyelination (Franklin and Ffrench-Constant, 2008). How to reconstitute the g-ratio and restore physiological function after remyelination still requires extensive research. To uncover how miR-200a&c protect against CNS demyelination, we performed metabolomic profiling on brain tissue from four groups—uninfected controls, AC-infected mice, infected mice receiving a negative control, and infected mice overexpressing miR-200a&c. This analysis enabled the identification of key transcription factors and signaling pathways mediating the observed protective effects. Metabolomic analysis confirmed that miR-200s altered protein expression at the cellular level. KEGG classification and enrichment analysis suggested potential pathways associated with the therapeutic effects of miR-200s, including those linked to stem cell pluripotency, cell adhesion molecules, and interactions with neuroactive light receptors. These results support the utility of miR-200a and miR-200c overexpression in treating AC-induced demyelination. To our knowledge, this is the first report of a treatment approach for demyelinating diseases. One study showed that the PI3K/Akt–HIF-1α pathway controls LDHA expression and thereby modulates neutrophil chemotaxis and phagocytosis. In sepsis, transcriptomic analyses revealed major shifts in glycolytic enzymes and the mTOR/HIF-1α axis, while metabolomic profiling demonstrated a pronounced shift toward aerobic glycolysis. Together, these results suggest that PI3K/Akt–HIF-1α–mediated downregulation of LDHA—and the resulting inhibition of glycolysis—drives neutrophil immunosuppression during sepsis (Pan et al., 2022). Therefore, based on the above studies and our metabolomics analysis results, we hypothesized that miR-200a&c exerts its protective effect of improving demyelination by regulating the PTEN/PI3K/AKT/mTOR pathway, so we conducted the following experiments.

We observed a negative correlation between the MBP and PTEN mRNA expression levels. PTEN inhibits axonal regeneration (Lin et al., 2025; Shi et al., 2025) and oligodendrocyte development (Lin et al., 2013; Gonzalez-Fernandez et al., 2018). Moreover, PTEN mutations have been identified in patients with multifocal demyelinating motor neuropathy (Bansagi et al., 2018). Our luciferase reporter gene assay validated PTEN as a target gene of miR-200a and miR-200c in OPCs. Inhibiting PTEN protein expression activated the downstream PI3K/AKT/mTOR signaling pathway, which is similar to the regulatory mechanism by which the miR-17-92 cluster promotes oligodendrocyte proliferation (Budde et al., 2010). We found that miR-200a and miR-200c protected *in vitro*-cultured OPCs from LPS-induced damage. PPAR-γ, which activates the PTEN signaling cascade, also induces OPC differentiation (Paintlia et al., 2010); however, Buller et al. (2012) reported that miR-200 targets serum reactive factor and negatively regulates OPC differentiation, hindering myelin regeneration. It has been reported that cannabinol reduces demyelination in EAE mice by activating the PI3K/Akt/mTOR pathway (Giacoppo et al., 2017). Our study provides additional evidence supporting the beneficial effects of PTEN inhibition. However, Harrington et al. (2010) found progressive myelin abnormalities and extensive axonal degeneration in myelin repair in a lysine-induced mouse model of demyelination when PTEN function was lost in the oligodendrocyte lineage. Hence, further studies are required to address this discrepancy.

Our study involved miR-200 overexpression in a mouse model of acute demyelination induced by AC infection and is the first observation of the protective effects of these miRNAs against demyelination. However, the study have some limitations. First, the effects of the miR-200s were only assessed in an AC infection–induced demyelination model, and its role in other classic demyelination models has yet to be validated. Therefore, further research is needed to determine whether similar therapeutic effects can be achieved in other models. Additionally, while our findings suggest that miR-200s promote myelination by targeting PTEN and activating the PI3K/Akt/mTOR pathway, we did not perform any functional experiments (such as loss-of-function or gain-of-function studies) to explore the precise mechanism. Further investigation using these techniques would strengthen our understanding of the mechanism of action of these miRNAs.

In conclusion, our findings confirm that the PTEN-PI3K/Akt/mTOR pathway mediates the protective effect of miR-200s against demyelination. Consequently, we propose miR-200 as a promising diagnostic biomarker of, and a novel therapeutic intervention for, demyelination-associated disorders. This treatment approach could help promote timely myelination among patients with demyelinating diseases.

## Additional files:

***Additional file 1:*** *AC antigen preparation and OPC stimulation experiment.*

Additional file 1 AC antigen preparation and OPC stimulation experiment

***Additional Figure 1:*** *miR-200a and -200c are beneficial for the survival of oligodendrocyte progenitor cells treated with AC antigen.*

Additional Figure 1 miR-200a and -200c are beneficial for the survival of oligodendrocyte progenitor cells treated with AC antigen.
Immunofluorescence of NG2-labeled (green) oligodendrocyte progenitor cells treated with AC antigen. Scale bar: 250 μm. *P < 0.05, *vs.* control group; ##*P* < 0.01, *vs.* AC-infected group. The data are presented as the means ± SEMs. AC: *Angiostrongylus cantonensis;* DAPI: 4',6-diamidino-2-phenylindole.

***Additional Figure 2:*** *Therapeutic effect of miR-200a and -200c on protein expression in the normal group.*

Additional Figure 2 Therapeutic effects of miR-200a and -200c on protein expression in the normal group.
(A) Principal component analysis (PCA) of metabolomic profiles across four groups: Normal (N), AC, ACNC, and AC-200ac. Each ellipse represents the 95% confidence interval of each group. (B) Radar chart showing the relative abundance of metabolites in the Normal (N) group. (C) Radar chart showing the relative abundance of metabolites in the AC-200ac treatment group.

## Data Availability

*All relevant data are within the paper and its Additional files*.
