# Supplementary material for: MicroRNA-200s attenuate demyelination caused by Angiostrongylus cantonensis in a mouse model by targeting phosphatase and tensin homolog
Source: Neural Regen Res. 2025 Jun 19;21(6):2599–608. doi: 10.4103/NRR.NRR-D-24-01112 (PMC13211809; doi:10.4103/NRR.NRR-D-24-01112)
Supplement: Supplementary file 1 [file NRR-21-2599_Suppl1.pdf]

Additional file 1

AC antigen preparation and OPC stimulation experiment

The brain tissues (lung, aorta, and heart) of the mice were dissected, and the mice were sacrificed 21 days after infection. The mixture was resuspended with PBS, and the supernatant was removed. This process was repeated many times until the insect body suspension was clean (if the shaking table was used, the nymphal body was shaken to the bottom of the centrifuge tube, the speed was reduced, or the sample was washed several times less). An equal volume of magnetic beads was added to the EP tube containing the worms. For 100 phase IV larvae, 400 μL of PBS and 4 μL of 100 mM PMSF (Beyotime) were added to a final concentration of 1 mM. The tissue was ground or homogenized at 4°C for 5 minutes in an ice bath. The mixture was then sonicated for 3 minutes, for a total of five times. Freezing and thawing were repeated three times. The mixture was subsequently centrifuged at 277 × g for 20 minutes at 4°C, after which the supernatant was collected. The bacteria were filtered through a 0.22 μm filter. The protein content was measured via the BCA method, and the samples were divided into multiple EP tubes. The mixture was stored at -80°C in a refrigerator.

To further study the repair effect of miRNA-200s on OPCs after stimulation with AC antigens, we pretreated OPCs with agomir-NC or agomir-miRNA for 24 hours and then stimulated the OPCs with the antigens for another 24 hours to test how this process works with stimulation time. The antigen concentration used in each well of the plate was 7 μg/μL, and the OPCs were grown uniformly in a 24-well plate.
