# Supplementary figures and images for: MicroRNA-200s attenuate demyelination caused by Angiostrongylus cantonensis in a mouse model by targeting phosphatase and tensin homolog
Source: Neural Regen Res. 2025 Jun 19;21(6):2599–608. doi: 10.4103/NRR.NRR-D-24-01112 (PMC13211809; doi:10.4103/NRR.NRR-D-24-01112)

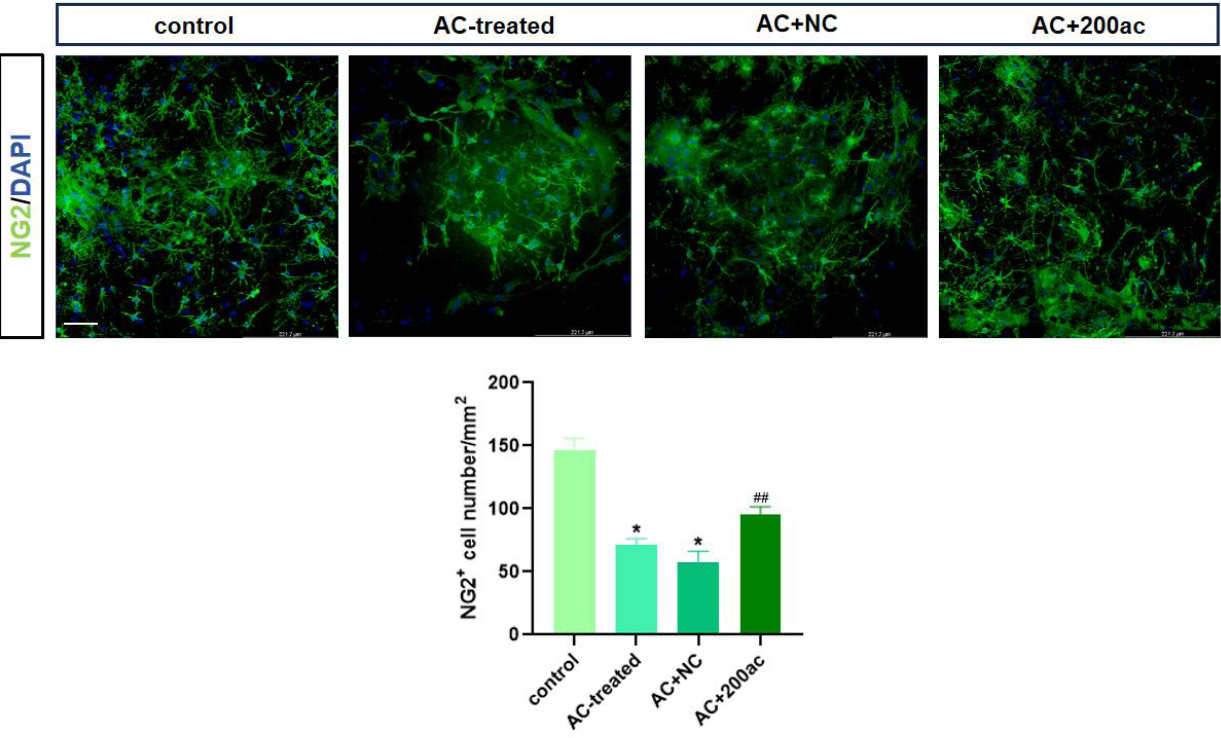

Supplement: Supplementary file 2 [file NRR-21-2599_Suppl1.tif]

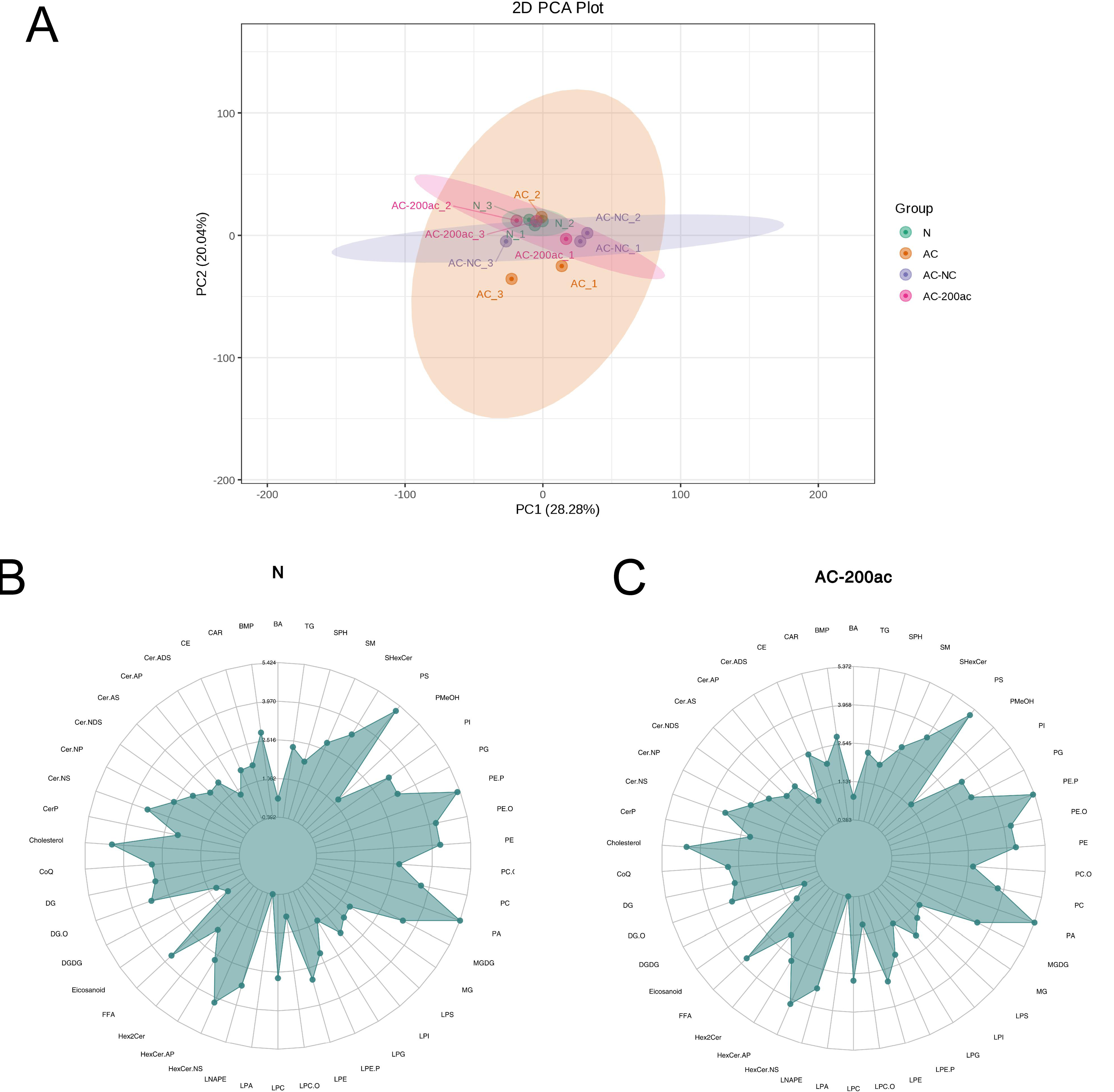

Supplement: Supplementary file 3 [file NRR-21-2599_Suppl2.tif]
